# Mass Cytometry Reveals the Imbalanced Immune State in the Peripheral Blood of Patients with Essential Hypertension

**DOI:** 10.1155/2023/9915178

**Published:** 2023-02-27

**Authors:** Rui Yang, Yuhong He, Honggang Zhang, Qiuju Zhang, Bingwei Li, Changming Xiong, Yubao Zou, Bingyang Liu

**Affiliations:** ^1^Institute of Microcirculation, Chinese Academy of Medical Sciences & Peking Union Medical College, Beijing 100005, China; ^2^State Key Laboratory of Cardiovascular Disease, Fuwai Hospital, National Center for Cardiovascular Diseases, Chinese Academy of Medical Sciences & Peking Union Medical College, Beijing 100037, China

## Abstract

Mounting evidence has confirmed that essential hypertension (EH) is closely related to low-grade inflammation, but there is still a lack of in-depth understanding of the state of immune cells in the circulating blood of patients with EH. We analyzed whether hypertensive peripheral blood immune cell balance was destroyed. The peripheral blood mononuclear cells (PBMCs) of all subjects were analyzed using time-of-flight cytometry (CyTOF) based on 42 kinds of metal-binding antibodies. CD45^+^ cells were categorized into 32 kinds of subsets. Compared with the health control (HC) group, the percentage of total dendritic cells, two kinds of myeloid dendritic cell subsets, one intermediate/nonclassical monocyte subset and one CD4^+^ central memory T cell subset in the EH group, was significantly higher; the percentage of low-density neutrophils, four kinds of classical monocyte subsets, one CD14^low^CD16^−^ monocyte subset, one naive CD4^+^ and one naive CD8^+^ T cell subsets, one CD4^+^ effector and one CD4^+^ central memory T cell subsets, one CD8^+^ effector memory T cell subset, and one terminally differentiated *γδ* T cell subset, decreased significantly in EH. What is more, the expression of many important antigens was enhanced in CD45^+^ immune cells, granulocytes, and B cells in patients with EH. In conclusion, the altered number and antigen expression of immune cells reflect the imbalanced immune state of the peripheral blood in patients with EH.

## 1. Introduction

According to statistics, the number of people aged 30-79 years with hypertension doubled to 1.28 billion worldwide in the 30 years from 1990 to 2019, while the treatment rate was less than half [1]. We know that essential hypertension (EH) of unknown etiology accounts for the majority of hypertension, even up to 95% [2]. Hypertension and cardiovascular diseases (CVD) are actually low-grade inflammatory diseases [3]. In hypertension, the self-perpetuating cycle of inflammation and oxidative stress is an important cause of vascular pathology and renal damage [4]. Hypertension induces dendritic cells (DCs) to produce reactive oxygen species (ROS) [5], which in turn stimulates DCs to produce IL-1*β*, IL-6, and IL-23, thereby promoting the polarization of T cells and producing IL-17A [6, 7]. An investigation showed that increased endothelial mechanical stretch promoted the conversion of monocytes to intermediate monocytes and stimulated monocytes to express IL-6, IL-1*β*, IL-23, and TNF-*α* [8]. Curiously, researchers found that resting neutrophils from patients with EH had increased CD11b and CD18 fluorescence intensity compared to healthy individuals, but the opposite results appeared in neutrophils activated with formyl-methionyl-leucyl-phenylalanine (fMLP) [9]. T cells have been reported to be reduced in the blood of hypertensive patients [10], and animal experiments have shown increased B cells in the spleen and kidney of hypertensive mice and IgG accumulation in the aortic membrane [11, 12]. Inflammation and abnormal activation of the immune system are now thought to be the important mechanisms in the progression of hypertension, which could be used to develop more effective therapies to alleviate end-organ damage in patients with hypertension [13].

What is the immune state of peripheral blood in EH patients? There is not a fully description yet. In this study, time-of-flight cytometry (CyTOF) was used to analyze various types of peripheral blood mononuclear cells (PBMCs) to more comprehensively assess the peripheral blood immune cell state in patients with EH.

## 2. Materials and Methods

### 2.1. Grouping

There were 5 patients with EH and 5 healthy controls (HCs). All subjects were male, and recruited from Fuwai Hospital, Chinese Academy of Medical Sciences & Peking Union Medical College (CAMS & PUMC; Beijing, China). The inclusion criteria for EH were in accordance with the 2018 Chinese guidelines for the management of hypertension, i.e., in the absence of antihypertensive drugs, the mean of three blood pressures measured on the nonsame day, systolic blood pressure (SBP) ≥ 140 mmHg and (or) diastolic blood pressure (DBP) ≥ 90 mmHg, was taken as the basis for hypertension. Diabetes, coronary disease, abnormal liver/renal function, classic chronic inflammatory diseases, secondary hypertension, and other serious diseases were excluded. The study protocol was approved by the Ethics Committee at the Institute of Microcirculation at the CAMS & PUMC and adhered to the tenets of the *Declaration of Helsinki* as well as applicable Chinese laws. Subjects were measured for blood pressure and risk factors associated with CVD; the basic information of the two groups is shown in Table 1.

### 2.2. Collection of PBMCs and Cell Staining

Detailed steps are shown in supplementary materials and methods, and 42 staining antibodies are shown in Table S1.

### 2.3. CyTOF Detection

Start the mass cytometry (Helios Mass Cytometry, FLUIDIGM), debug instruments and control quality standards (Tuning Solution, FLUIDIGM), the same number of cells in each sample were mixed together, add calibration beads (EQ™ Four Element Calibration Beads, FLUIDIGM), set channel name, data name, collection speed, and total cell number, and collect the raw data.

### 2.4. Data Analysis

The data was processed using the bead normalization method. Sample-specific barcodes were used to distinguish data for each individual in multiple sample data. Gates were used to distinguish live cells and immune cells and exclude debris, cell clumps, and beads (FlowJo v10.0.7). The *x*-shift clustering algorithm was used to process the date of markers in the panel. Then, the data was downsampled. Data-driven clustering analysis was used to obtain different cell clusters. 300,000 cells in each group were analyzed.

The specified cells were selected, and the dimensionality of the data was reduced by the *t*-distributed stochastic neighbor embedding (T-SNE) data visualization method. The viSNE map and heat map were generated according to the T-SNE coordinates, and the cell cluster numbers above, the percentages of cell clusters, and the expression intensities of metal-connected antibodies were shown. The sample information was grouped, box plots were made using the cluster-percentage matrix, and the average expression levels of the markers were displayed in the violin plots. The normality of the data was assessed using the Shapiro-Wilk test firstly, then unpaired two-tailed *t*-test, or Welch's *t*-test, or Mann–Whitney test was used to compare the difference between the two groups (*α* = 0.05). Pearson or Spearman correlation analysis was used to calculate the correlation between the index with significant difference between the two groups and blood pressure (GraphPad Prism 9.4.0).

## 3. Results

### 3.1. Basic Information of Subjects

The basic situation of the HC and EH groups is shown in Table 1. In our study, SBP, DBP, and risk factors for CVD such as body mass index (BMI), total cholesterol (TC), and low-density lipoprotein (LDL) values were significantly increased in EH compared to HC. Therefore, we also analyzed the direct correlation between some important indicators and blood pressure (the main diagnostic standard of hypertension). In addition, the influence of higher age in the EH group was analyzed and discussed later.

### 3.2. Analysis of PBMCs Using CyTOF

**Figure 1:**
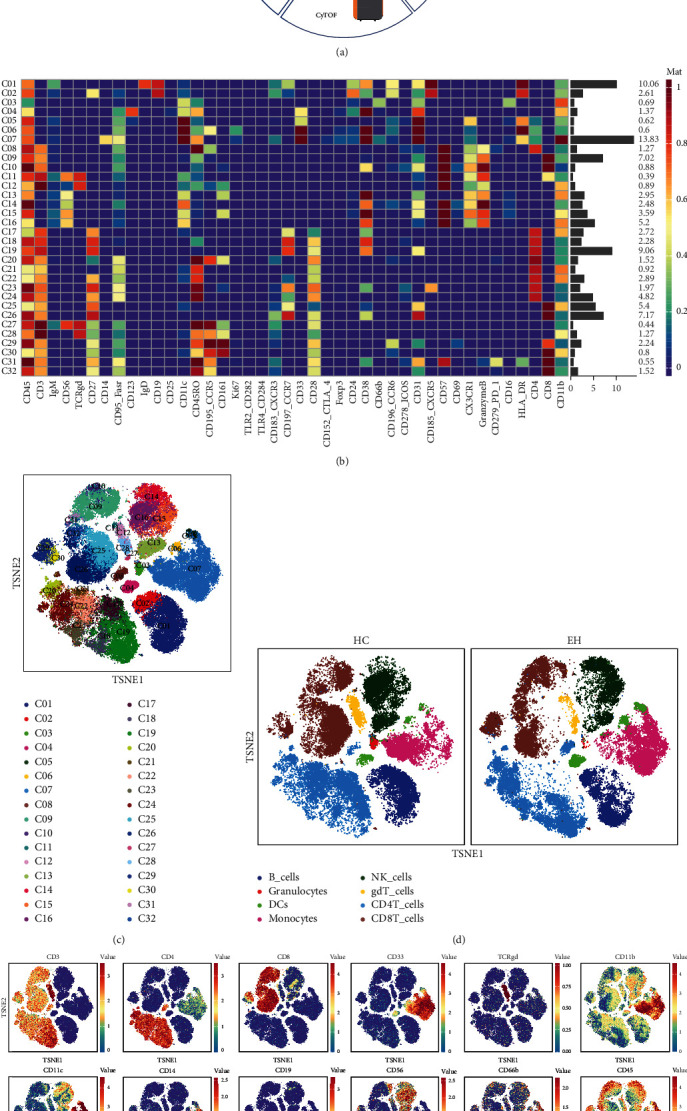
Single-cell mass cytometry analysis of peripheral blood samples and data visualization. (a) The experimental flow chart. (b) Heat map of “clusters vs. markers.” The percentage of each cell cluster is shown on the right of the heat map. The median value of each marker expression in each cell cluster is indicated by the shade of color. (c) Dimensional reduction visualization of merge samples. The 32 cell clusters are distributed in the T-SNE plot, marked with different colors. (d) T-SNE plots of cell subsets from HC and EH. Different colors indicate different types of cells. (e) The distribution of cell lineage markers in different lineage cells.

The experimental flow chart is shown in Figure 1(a). The merge sample data is processed by the T-SNE data visualization method, and 32 cell clusters are automatically formed in heat map and viSNE graph; all clusters are classified into different cell types (Figures 1(b)–1(d) and Table 2). The expression of a part of the cell lineage markers is shown in Figure 1(e), and they are clearly distributed among cells of different lineages.

### 3.3. Comparison of Cell Percentage and Marker Expression in CD45^+^ Cells between the HC and EH Groups

**Figure 2:**
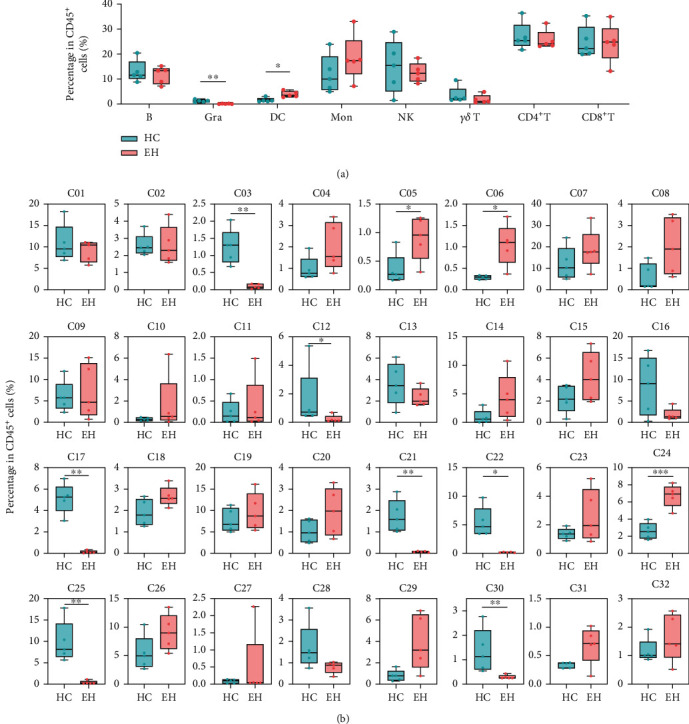
The percentage of different cell subsets in the HC and EH groups. (a) Percentage comparison of DCs, monocytes, granulocytes, NKs, CD4^+^ T cells, CD8^+^ T cells, *γδ* T cells, and B cells between the two groups. (b) The percentage difference of 32 cell subsets between the two groups. ^∗^*P* < 0.05, ^∗∗^*P* < 0.01, ^∗∗∗^*P* < 0.001.

The quantity of different immune cell subsets was compared between the HC and EH groups (Figure 2(a)). Compared with the HC group, the percentage of DCs was significantly increased in EH (*P* < 0.05), and the percentage of monocytes was increased but not significantly different (*P* = 0.25). Interestingly, granulocytes in the PBMC layer of EH were decreased (*P* < 0.01). The results showed no change in the percentage of natural killer cells (NKs) between the HC and EH groups (*P* = 0.64). In our study, there were no differences in the total number of CD4^+^ T cells (*P* = 0.42), CD8^+^ T cells (*P* = 0.93), and *γδ* T cells (*P* = 0.22) between the HC and EH groups. The percentage of B cells also showed no significant difference between the two groups (*P* = 0.50).

The average expression level of all markers in CD45^+^ cells of the HC and EH groups is shown in Figure S1, and CD45, CD95, CD45RO, CCR5, Ki67, TLR2, Foxp3, CD38, CD69, PD-1 were increased in EH, while CD11b was decreased. The functions of CD45, CD45RO, and CD11b are described in Table S2.

### 3.4. Characteristics of DCs in EH

DCs are generally divided into myeloid DCs (mDC, expressing CD11c and CD33) and plasmacytoid DCs (pDC, expressing CD123) [14]. In this study, DCs were subdivided into three cell subsets by clustering analysis (Figure 1(b)). Cluster 4 cells were defined as pDCs because of their high expression of CD123 [15], and compared with the HC group, there was no difference in the number of them in the EH group (Figure 2(b)). CX3CR1 (CX3C chemokine receptor 1) is the marker of mDCs [16], so CX3CR1^+^CD38^−^CD11c^+^HLA-DR^+^ DCs (cluster 5) were defined as mDCs here; we also defined Ki67^+^CD11c^+^HLA-DR^+^CD123^−^ DCs (cluster 6) as proliferative mDCs [14, 15, 17], it is also characterized as CCR5^+^, which is usually expressed on immature DCs [18]. As the results showed, the two kinds of mDCs were both increased in EH (*P* < 0.05, *P* < 0.05, respectively; Figure 2(b)).

### 3.5. Characteristics of Monocytes in EH

Monocytes in peripheral blood are usually divided into three subsets: CD14^++^CD16^−^ (classical), CD14^++^CD16^+^ (intermediate), and CD14^+^CD16^++^ (nonclassical), which are present in 80-95%, 2-8%, and 2-11% of circulating monocytes, respectively [19–21]. Intermediate monocytes and nonclassical monocytes expand under inflammatory condition, and the phosphorylation level of STAT3 was higher in intermediate monocytes than in the other two types of monocytes [8]. Typically, CX3CR1 is highly expressed in intermediate and nonclassical monocytes, and HLA-DR is highly expressed on intermediate monocytes [20].

**Figure 3:**
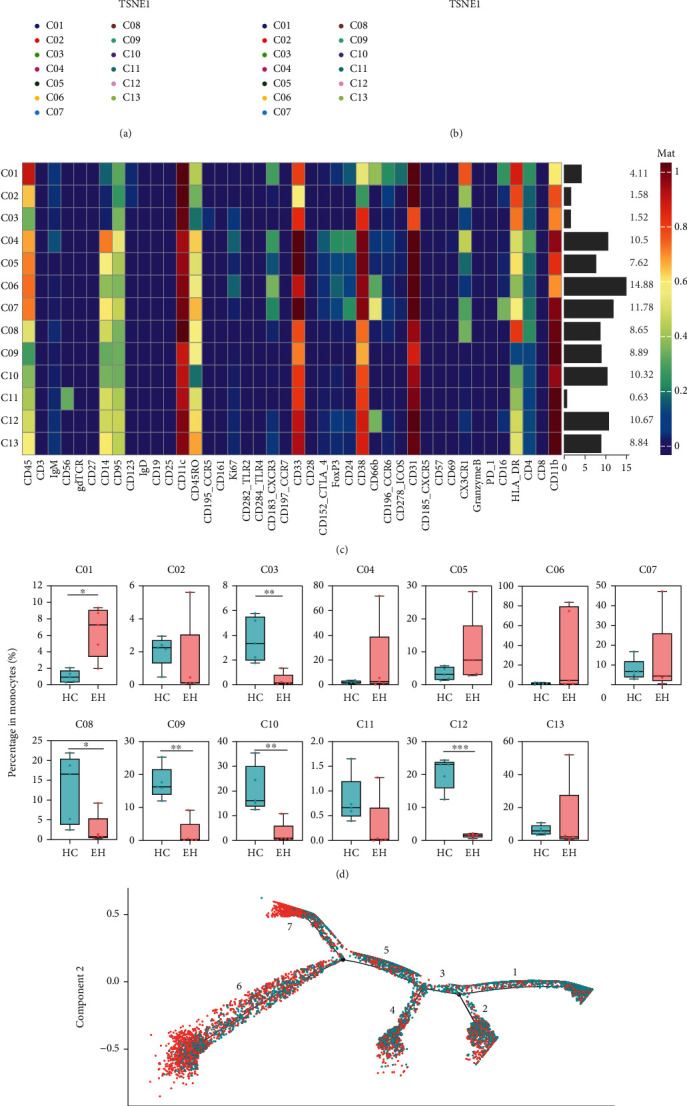
Difference of monocytes between the HC and EH groups. (a) Second clustering analysis of monocytes (merge). (b) T-SNE plots of 13 monocyte subsets of the HC and EH groups. (c) The heat map of monocytes (merge). (d) Difference in the percentage of monocyte subsets in HC and EH. (e) The plot of the pseudotime analysis for the HC and EH groups. There are 3 branch points and 7 branches in this picture. ^∗^*P* < 0.05, ^∗∗^*P* < 0.01, ^∗∗∗^*P* < 0.001.

In this research, the only cluster of monocytes (cluster 7; Figure 1(b)) was further divided into 13 cell subsets (Figures 3(a)–3(c)). The results showed an increased percentage of CD45^hi^CXCR3^+^CX3CR1^+^HLA-DR^hi^CD11b^low^CD14^+^CD16^+^ monocytes (cluster 1), which expresses lower CD14 and higher CD16 like intermediate/nonclassical monocytes, decreased percentage of CD14^low^CD16^−^ monocytes (cluster 3), and decreased percentage of CD45^low^CXCR3^−^CD14^+^CD16^-/low^ monocytes (clusters 8, 9, 10, and 12) similar to classical monocytes in EH (*P* < 0.05, *P* < 0.01, *P* < 0.05, *P* < 0.01, *P* < 0.01, and *P* < 0.001, respectively; Figure 3(d)).

Moreover, two markers, CD14 and CD16, were selected for the pseudotime analysis of monocytes in 13 clusters (Figures S2a and S2b). The results showed that the part of monocytes from patients with EH differentiated toward state 6 and state 7 (Figure 3(e)). And the pseudotime plots showed that the two states were CD14^hi^, and the expression of CD16 gradually increased from state 7 to state 6.

### 3.6. Characteristics of Granulocytes in EH

Neutrophils account for 70% of the peripheral blood circulation, which are first recruited to the site of inflammation [22]. In this study, interestingly, the results showed that PBMCs contained a small number of cells characterized as CD11c^+^CD66b^+^CD31^+^CD16^+^CD11b^+^ (cluster 3) (Figure 1(b)), and the number of these cells was significantly reduced in EH (*P* < 0.01; Figure 2(b)). Antigens CD66b, CD16, and CD11b were reported as markers of activated neutrophils [23]. Usually, granulocytes do not appear in the PBMCs but in the upper layer of the red blood cells (normal-density neutrophils, NDNs) when separating by Ficoll density gradient centrifugation method. Neutrophils in the PBMC layer, known as low-density neutrophils (LDNs), have been identified in systemic lupus erythematosus, rheumatoid arthritis, sepsis, asthma, natural pregnancy, tumors [22], COVID-19 [24], and arterial hypertension [25], but the function of LDNs in EH is not yet well defined.

**Figure 4:**
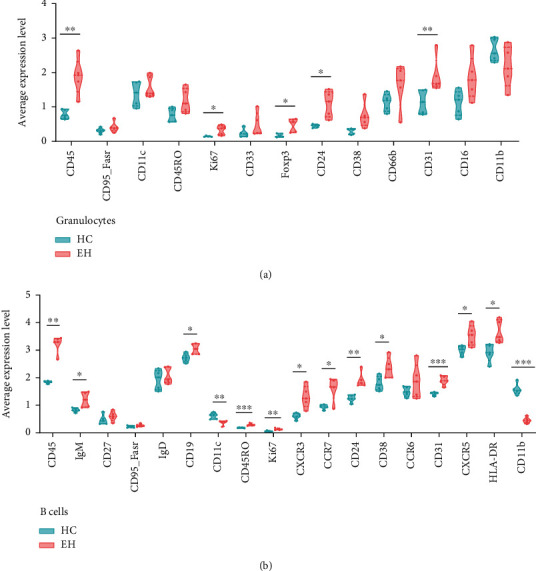
Difference of granulocytes and B cells between the HC and EH groups. (a) The difference in the average expression level of antigens in granulocytes of the HC and EH groups. (b) The difference in the average expression level of antigens in B cells of the HC and EH groups. ^∗^*P* < 0.05, ^∗∗^*P* < 0.01, ^∗∗∗^*P* < 0.001.

In addition, the average expression of antigens in the only granulocyte cluster of the two groups was compared, and most of the proteins in the EH group showed an increasing trend except CD11b (Figure 4(a)). The expression level of CD45, Ki67, Foxp3, CD24, and CD31 was significantly increased in the EH group, and their functions are shown in Table S2.

### 3.7. Characteristics of NKs in EH

By clustering analysis, four NK cell clusters were obtained (Figure 1(b)). Compared to HC, there was no significant change in the number of NKs in EH (Figures 2(a) and 2(b)).

### 3.8. Characteristics of T Cells in EH

CD4^+^ T cells, CD8^+^ T cells, and *γδ* T cells are closely associated with the development of hypertension [26]. Usually, CD45RO is used as a marker of activated/memory T cells [27], CD45RO^−^/CD45RA^+^ is used to distinguish naive T cells [28, 29]. Memory T cells can be further divided into three types based on the lymph node homing receptors CD62L and CCR7 [30, 31]. Central memory T cells (T_CM_, CD62L^high^CCR7^+^) are mainly stored in secondary lymphoid organs and have a high-proliferative capacity when reactivated [32]. Effector memory T cells (T_EM_, CD62L^low^CCR7^−^) exist in the periphery and can be rapidly recruited to inflammatory sites and produce effector factors [32, 33]. And resident memory T cells (T_RM_, CD62L^low^CCR7^−^CD69^+^) remain at the site of infection in order to provide more rapid and direct protection [32].

#### 3.8.1. CD4^+^ T Cells

In this study, nine CD4^+^ T cell clusters were identified (Figure 1(b)), and four of them showed significant difference in quantity between the HC and EH groups (Figure 2(b)). Cluster 17 was CD45RO^−^CCR7^+^CD95^−^ naive CD4^+^ T cell subset [28], and the percentage of it was significantly reduced in EH (*P* < 0.01). Cluster 21 cells characterized by CD45RO^+^CCR7^−^CD27^−^CD28^+^CD4^+^ were defined as effector memory T cells, which were decreased in EH (*P* < 0.01). Furthermore, two kinds of central memory CD4^+^ T cells, CD45^low^CD45RO^+^CCR7^+^CD27^+^CD28^+^CD4^+^ T cells (cluster 22) and CD45^hi^CD45RO^+^CCR7^+^CD27^+^CD28^+^CD4^+^ T cells (cluster 24), expressed similar antigens. However, the results showed that the number of cluster 22 cells with CD45^low^ decreased in EH, while the number of cluster 24 cells with CD45^hi^ increased (*P* < 0.05, *P* < 0.001, respectively).

#### 3.8.2. CD8^+^ T Cells

CD8^+^ T cells were clustered into eight clusters here (Figure 1(b)); the percentage of clusters 25 and 30 was significantly different between the two groups, while other clusters were not (Figure 2(b)). The results showed that CD45RO^−^CCR7^+^CD95^−^ naive CD8^+^ T cells (cluster 25) and CD45^low^CD45RO^low^CD161^+^CCR7^−^CCR6^low^CCR5^+^CD8^+^ effector memory T cells (cluster 30) [29], were both decreased in EH (*P* < 0.01, *P* < 0.01, respectively).

#### 3.8.3. *γδ* T Cells

As the results showed, *γδ* T cells were divided into four cell subsets (Figure 1(b)). Cluster 12 cells characterized by CD45^low^CD27^−^CD45RO^low^CD57^hi^Granzyme B^+^ were defined as terminally differentiated *γδ* T cells, and their proportion was reduced in EH (*P* < 0.05, Figure 2(b)).

### 3.9. Characteristics of B Cells in EH

In this study, B cells were divided into two subsets (Figure 1(b)), and the number of B cells was not altered in EH. However, compared to HCs, the expression of most markers in B cells from EH patients was significantly increased, including CD45, IgM, CD19, CD45RO, Ki67, CXCR3, CCR7, CD24, CD38, CD31, CXCR5, and HLA-DR (Figure 4(b) and Table S2).

### 3.10. The Correlation between Blood Pressure/Age and Immune Cells

After obtaining the indicators with significant differences between the two groups, we then analyzed their correlation with blood pressure (SBP, DBP, and pulse pressure difference (PP)) and other influencing factor (age), as shown in Table S3.

## 4. Discussion

### 4.1. The Increased Total DCs and mDCs in EH

In general, mDCs mainly produce the cytokines IL-1*β*, IL-6, IL-12, IL-23, and present soluble antigens; pDCs mainly produce type I and type III interferons to combat the virus [14]. CX3CL1, the ligand of CX3CR1, is expressed by smooth muscle cells, activated endothelial cells, epithelial cells, and neurons [34]. CX3CL1/CX3CR1 axis has both chemotaxis and adhesion capacity [35]. It was reported that CX3CR1 on DCs is a kidney-specific “homing receptor” [36]. CD38 is a nicotinamide dinucleotide (NAD^+^) catabolic enzyme that catabolizes NAD^+^ into adenosine diphosphate ribose (ADPR) and cyclic ADPR, which leads to calcium mobilization [37, 38]. It was shown that the migration of Cd38^−/−^DCs to lymph nodes was blocked and that the inhibition of CD38 attenuated the chemotaxis of DC cells towards CCL21 (a ligand of CCR7) [39, 40]. Therefore, myeloid CX3CR1^+^CD38^−^CD11c^+^HLA-DR^+^ DCs may have the ability to be recruited to the kidney rather than the lymph nodes. Under the physiological condition, immature DCs are mainly responsible for capturing autoantigens to induce immune tolerance; when inflammation occurs, they mature and activate T cells into effector cells [41]. Immature DCs reach the site of inflammation via CCR5, recognize the antigen, and become mature, subsequently triggering a T cell response [42]. The increased Ki67^+^CD11c^+^HLA-DR^+^CD123^−^ mDCs may represent an overactivated immune response in hypertension.

In addition, we found a small number of Foxp3^+^ DCs in peripheral blood, which may have immunosuppressive effects [43–45], but currently, there are few relevant studies, which deserve more attention.

### 4.2. The Imbalanced State of Monocytes in EH

CD14^+^CD16^+^ monocytes have a strong capacity to present antigen due to HLA-DR and promote TNF secretion [46]. As mentioned above, CD45^hi^CXCR3^+^CX3CR1^+^HLA-DR^hi^CD11b^low^CD14^low^CD16^+^ monocytes, which are similar to intermediate/nonclassical monocytes, increased in EH, are proinflammatory, and the expression of chemokines may make them easier to migrate to tissues [34, 47, 48]. On the contrary, the four subsets of classical monocytes in the EH group almost disappeared. In conclusion, the changes of monocytes may all indicate enhanced immune activity in EH.

### 4.3. Abnormal State of LDNs in EH

Neutrophils exert their antimicrobial action through phagocytosis, degranulation, and extracellular traps [22]. When inflammation occurs, endothelial cells are activated to express adhesion molecules that promote neutrophil adhesion [22]. Curiously, in our study, LDNs from hypertensive patients were reduced but with higher expressed antigens (except CD11b) compared to controls. Coincidentally, a recent study showed that patients with hypertension have a higher proportion of NDNs compared to healthy individuals [25]. Is there a correlation between LDNs and NDNs? And how? We are not sure whether LDNs are developing immature neutrophils or degranulated neutrophils, and their pathophysiological mechanism in hypertension deserves more exploration.

### 4.4. Varied T Cell Subsets in EH

Total CD4^+^ T cells and total CD8^+^ T cells have been reported to be reduced in hypertension [10]. And in our study, the results showed no significant change in their respective total number, but the subsets of CD4^+^, CD8^+^, and *γδ* T cells were altered in EH. We found that CD4 and CD8 were not only expressed on CD4^+^ T cells and CD8^+^ T cells, but also on *γδ* T cells, NKs and DCs. So, different classification standards may be the reasons for the different results.

When inflammation occurs, naive T cells are activated by antigen presented by antigen-presenting cells (APCs), beginning to proliferate and produce effector T cells. When inflammation disappears, small number of effector cells survive and become antigen-specific memory cells [30, 49]. Normally, naive T cells in the blood enter secondary lymphoid organs for patrol and can proliferate rapidly when activated [49]. Our results showed that the percentage of CD45RO^−^CCR7^+^CD95^−^ naive CD4^+^ T cell subset and CD45RO^−^CCR7^+^CD95^−^ naive CD8^+^ T cell subset is reduced in the EH group. DCs can activate naive T cells into effector cells [50]; in our results, increased DCs may also explain the reduced number of naive T cells. It has been reported that naive T cells decrease and memory T cells increase with age [51, 52], but the difference between 22 and 40 years old was not emphasized in those studies. And in this study, the results showed that the decrease of naive CD4^+^ and naive CD8^+^ T cells was also associated with increasing age.

In our study, CD4^+^ and CD8^+^ memory T cells were divided into multiple subsets and had different changes. CD27 and CCR7 can mediate immune cells homing to lymph nodes; CD4^+^ and CD8^+^ T cells expressing CCR7 usually also express CD27 and CD28 (a costimulatory molecule for T cell activation), but not vice versa [29, 53]. And CCR7^−^CD45RA^−^CD27^−^CD28^+^CD4^+^ T cells are effector memory T cells that secrete IFN-*γ*, IL-4, and IL-2 [54]. Meanwhile, the investigators defined CCR7^+^CD45RA^−^CD27^+^CD28^+^CD4^+^ T cells as central memory T cells with a weaker ability to produce cytokines [54]. CD161^+^CD8^+^ T cells have the ability to secrete IL-17 and cytotoxicity [55, 56]. In addition, the researchers found increased infiltration of CD161^+^ immune cells in the spleen, kidney, and aorta of spontaneously hypertensive rats (SHR) and that IL-17 secreted by CD161^+^ cells mediated endothelial injury and hypertension [57]. CCR6 mediates cell migration through the ligand CCL20, which is usually highly expressed in tissues such as intestinal mucosa, lung mucosa, liver, and skin [58]. Above all, the reduction of CD45^low^CD45RO^low^CD161^+^CCR7^−^CCR6^low^CCR5^+^CD8^+^ effector memory T cells in hypertension may be due to tissue infiltration or cell transformation.

In general, *γδ* T cells are CD4^−^CD8^−^CD3^+^ cells that can bind antigens without the major histocompatibility complex (MHC) [59]. They can produce IFN-*γ* and IL-17 in infection [60, 61] and have a strong proinflammatory effect [62]. And CD45^low^CD27^−^CD45RO^low^CD57^hi^Granzyme B^+^*γδ* T cells were defined as terminally differentiated cells with low proliferative and high effector capacity [59, 63, 64].

### 4.5. Enhanced B Cell Antigens in EH

B cells play an important role in humoral immunity by secreting antibodies and forming memory cells [65]. But the role of B cells in hypertension is still not very clear [10, 66]. Our results showed no significant difference in the number of B cells between the HC and EH groups, which was consistent with the previous study [10]. But the expression of most B cell antigens, CD45, IgM, CD19, CD45RO, Ki67, CXCR3, CCR7, CD24, CD38, CD31, CXCR5 and HLA-DR which affect the function of B cells, is significantly increased in EH. In summary, it is suggested that the activation, migration, and immune effects of B cells may be correspondingly augmented. Since we did not detect cytokines and soluble antibodies here, we were unable to further analyze the role of humoral immunity of B cells in hypertension.

## 5. Conclusion

**Figure 5:**
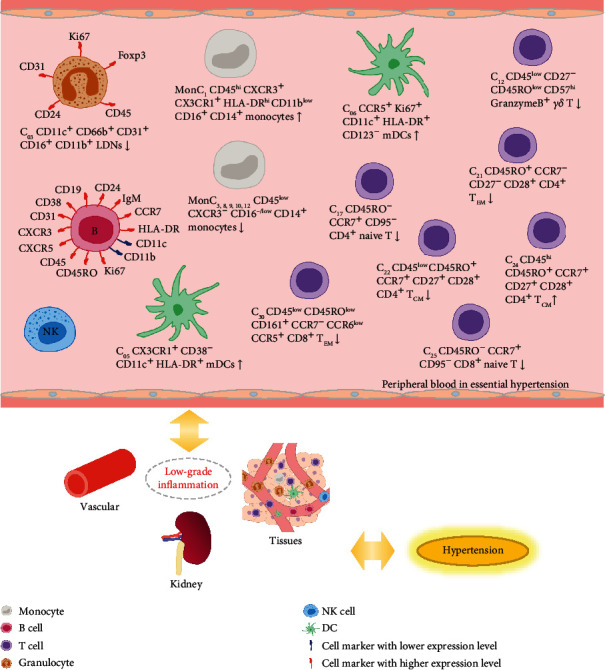
Summary and inference. The unbalanced immune state may be associated with the occurrence and development of hypertension by affecting blood vessels, kidneys, and tissues closely related to hypertension. Maintaining the balance of immune status may be an important direction for improving essential hypertension.

The summary of this study is shown in Figure 5. Our results showed that the expression of many important antigens was significantly different in CD45^+^ PBMCs of hypertensive patients, and many characteristic antigens in granulocytes and B cells were enhanced; they may serve as characteristic antigens for the development of EH. The increased percentage of total DCs and two mDC subsets emphasized the distinct role of DCs in hypertension. The number of proinflammatory monocytes and normal monocytes in EH is unbalanced. LDNs are reduced in EH, and more research is needed to determine their origin and function. The percentage of the one naive CD4^+^ and one naive CD8^+^ T cell subsets was decreased in EH. However, the changes of memory T cells are diverse. In conclusion, our findings provide a more comprehensive description for the imbalanced immune state and inflammatory environment in peripheral blood of patients with EH, and larger sample size is needed in the future. The detailed mechanisms of altered immune cell state in EH require more research.

## Figures and Tables

**Table 1:** Basic characteristics of objects.

Index parameter	The HC group (*n* = 5)	The EH group (*n* = 5)	*P* value
Age (years)	23.2 ± 0.8	35.2 ± 4.9	0.005
Gender	NA	NA	
Systolic blood pressure (mmHg)	119.4 ± 7.3	157.0 ± 15.6	0.001
Diastolic blood pressure (mmHg)	73.4 ± 2.4	101.4 ± 10.2	0.003
Pulse pressure difference (mmHg)	46.0 ± 5.3	55.6 ± 15.9	0.24
Body mass index (kg/m^2^)	24.3 ± 2.3	29.1 ± 2.4^a^	0.03
Total cholesterol (mmol/L)	3.8 ± 0.8	5.3 ± 0.7^b^	0.02
Low-density lipoprotein (mmol/L)	2.2 ± 0.5	3.5 ± 0.6^b^	0.01
High-density lipoprotein (mmol/L)	1.5 ± 0.4	1.2 ± 0.2^b^	0.19
Triglycerides (mmol/L)	0.6 ± 0.3	2.3 ± 1.6^b^	0.14

Note: the value of each parameter is expressed as the mean ± SD. *P* value obtained by comparing the mean of two groups of random samples with *t*-test or Mann–Whitney test; NA was not available (because all subjects are men). ^a^*n* = 3; ^b^*n* = 4.

**Table 2:** Cell classification.

Cell type	Marker	Cell type	Marker
DCs	CD11c^+^HLA-DR^+^CD123^+/-^	CD4^+^ T cells	CD3^+^CD4^+^
Granulocytes	CD66b^+^CD16^+^CD11b^+^	CD8^+^ T cells	CD3^+^CD8^+^
Monocytes	CD14^+^	*γδ* T cells	CD3^+^*γδ*TCR^+^
NKs	CD3^−^CD56^+^	B cells	CD19^+^

## Data Availability

The statistical data that support the findings of this study are available from the corresponding authors upon reasonable request.

## Abbreviations

APC: Antigen-presenting cell
CCL: C-C motif chemokine ligand
CCR: C-C motif chemokine receptor
CD: Cluster of differentiation
CX3CR1: C-X3-C motif chemokine receptor 1
CyTOF: Cytometry by time of flight
EH: Essential hypertension
Foxp 3: Forkhead box protein 3
HC: Healthy control
IL: Interleukin
LDN: Low-density neutrophil
mDC: Myeloid dendritic cell
NDN: Normal-density neutrophil
PBMC: Peripheral blood mononuclear cell
pDC: Plasmacytoid dendritic cell
T_CM_: Central memory T cell
T_EM_: Effector memory T cell
T_RM_: Resident memory T cell
T-SNE: *T*-distributed stochastic neighbor embedding.

## Supplementary Materials

Supplementary Materials Supplementary materials and methods. Table S1: CyTOF staining panel of antibodies. Figure S1: the difference in the average expression level of 42 kinds of markers in CD45^+^ cells between the HC and EH groups. Table S2: markers in EH. Figure S2: pseudotime analysis of monocytes (merge sample). (a) The pseudotime analysis of CD14. (b) The pseudotime analysis of CD16. Table S3: the correlation between indexes and blood pressure/age.
