# Supplementary material for: Mass Cytometry Reveals the Imbalanced Immune State in the Peripheral Blood of Patients with Essential Hypertension
Source: Cardiovasc Ther. 2023 Feb 27;2023:9915178. doi: 10.1155/2023/9915178 (PMC9988372; doi:10.1155/2023/9915178)
Supplement: Supplementary Materials — and methods. Table S1: CyTOF staining panel of antibodies. Figure S1: the difference in the average expression level of 42 kinds of markers in CD45+ cells between the HC and EH groups. Table S2: markers in EH. Figure S2: pseudotime analysis of monocytes (merge sample). (a) The pseudotime analysis of CD14. (b) The pseudotime analysis of CD16. Table S3: the correlation between indexes and blood pressure/age. [file 9915178.f1.pdf]

Supplementary Materials and Methods

Collection of PBMCs

10 mL of EDTA whole blood (diluted 1:1) with PBS (Phosphate-Buffered Salin, GENOM) was carefully layered on a 50 mL tube of ficoll separation solution (Ficoll-Paque PLUS, GE Healthcare) and centrifuged at 400 g for 15 min with '1' brake at 20 °C. The cell interface white cloudy layer was carefully separated into 15 mL tubes and washed with 10 mL PBS for 10 min at 400 g 20 °C, discarding the supernatant and repeating this once. Cells were re-suspended with PBS for counting and placed on ice.

Cell Phenotype Panel

42 protein markers were used to distinguish immune cell subsets, the CyTOF staining panel antibodies detected in this study are shown in Table S1.

Cell Staining

Cell viability staining: 3×10^6 PBMCs were taken from one sample, resuspend and stained with 100 μL 0.25 μM 194Pt staining agent (Cell-ID™ Cisplatin-194Pt, FLUIDIGM), incubated on ice for 5 min, washed 2 times with the FACS buffer for 5 min at 400 g 4 °C. **Blockade of non-specific Fc receptors:** The cells were resuspended with 50 μL prepared blocking solution and incubated on ice for 20 min. **Extracellular staining:** 50 μL extracellular antibody mixture was pre-prepared and added directly to the sample containing the blocking mixture, incubated on ice for 30 min, and the cells were washed twice with the FACS buffer for 5 min at 400 g 4 °C. **DNA staining:** The cells were resuspended in 200 μL 250 nM Ir staining agent (Cell-ID™ Intercalator-Ir, FLUIDIGM) diluted with fix and perm buffer (Maxpar® Fix and Perm Buffer, FLUIDIGM) and incubated overnight at 4°C.

Intracellular staining: Cells were washed twice with 1mL 1x permeabilization buffer (eBioscience™ Permeabilization Buffer (10x), Thermo Fisher Scientific) for 5 min at 800 g 4 °C, and fixed with 100 μL diluted fixation/permeabilization solution ($V_{diluent}$: $V_{concentrate}$ = 3 : 1) at room temperature for 30 min, washed twice with 1x permeabilization buffer for 5 min at 800 g 4 °C, added 100 μL intracellular antibody mixture, and incubated on ice for 30 min. **Sample coding labeling:** Cells were washed with FACS buffer, centrifuged, resuspended with barcode reagent, and incubated on ice for 20 min. **Cleaning the cells before operation:** Cells were washed with FACS buffer, centrifuged for 5 min at 800 g 4 °C, resuspended with dd H_2O, and filtered by a flow cytometry screen for cell counting and CyTOF detection.

TABLE S1: CyTOF staining panel of antibodies

Li	Label	Marker	Clone	Dilution	Brand
**1**	89Y	CD45	HI30	100	BioLegen
**2**	115In	CD3	UCHT1	200	BioLegen
**3**	139La	IgM	MHM-88	50	BioLegen
**4**	141Pr	CD56	NCAM16.2	400	BD
**5**	142Nd	TCRgd	5A6.E9	200	PLT
**6**	143Nd	CD27	O323	200	BioLegen
**7**	144Nd	CD14	M5E2	50	BioLegen
**8**	145Nd	CD95(Fasr)	DX2	100	BioLegen
**9**	146Nd	CD123	6H6	400	BioLegen
**10**	147Sm	IgD	IA6-2	800	BioLegen
**11**	148Nd	CD19	HIB19	200	BioLegen
**12**	149Sm	CD25	24212	200	RD
**13**	150Nd	CD11c	BU15	100	BioLegen
**14**	151Eu	CD45RO	UCHL1	50	BioLegen
**15**	152Sm	CD195 (CCR5)	J418F1	100	BioLegen
**16**	153Eu	CD161	HP-3G10	25	BioLegen
**17**	154Sm	Ki67	SolA15	400	eBioscienc
**18**	155Gd	TLR2 CD282	TL2.1	25	Biolegend
**19**	156Gd	TLR4 CD284	HTA125	25	BioLegen
**20**	157Gd	CD183(CXCR3)	G025H7	50	BioLegen
**21**	158Gd	CD197(CCR7)	G043H7	50	BioLegen
**22**	159Tb	CD33	WM53	800	BioLegen
**23**	160Gd	CD28	CD28.2	50	BioLegen
**24**	161Dy	CD152(CTLA-4)	14D3	200	eBioscienc
**25**	162Dy	Foxp3	PCH101	50	eBioscienc
**26**	163Dy	CD24	ML5	50	BioLegen
**27**	164Dy	CD38	HIT2	50	BioLegen
**28**	165Ho	CD66b	G10F5	200	BioLegen
**29**	166Er	CD196(CCR6)	G034E3	100	BioLegen
**30**	167Er	CD278(ICOS)-h/m	C398.4A	200	BioLegen
**31**	168Er	CD31	WM59	200	BioLegen
**32**	169Tm	CD185(CXCR5)	RF8B2	100	BD
**33**	170Er	CD57	HNK-1	800	BioLegen
**34**	171Yb	CD69	FN50	200	BioLegen
**35**	172Yb	CX3CR1	K0124E1	200	BioLegen
**36**	173Yb	Granzyme B	GB11	100	Fluidigm
**37**	174Yb	CD279/PD-1	EH12.2H7	100	BioLegen
**38**	175Lu	CD16	3G8	800	BioLegen
**39**	176Yb	HLA-DR	L243	200	BioLegen
**40**	197Au	CD4	RPA-T4	800	BioLegen
**41**	198Pt	CD8a	RPA-T8	400	BioLegen
**42**	209Bi	CD11b	M1/70	800	BioLegen

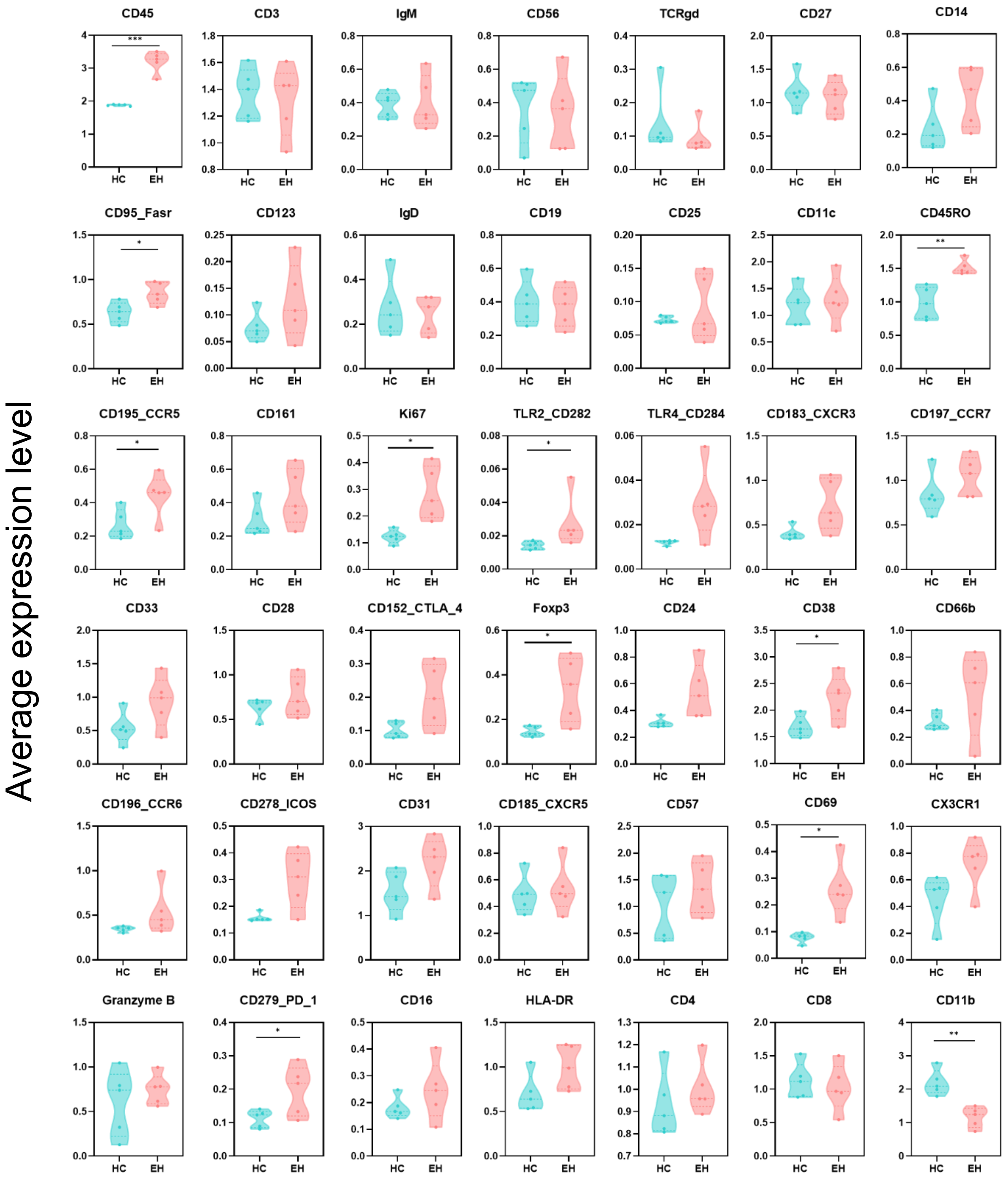

FIGURE S1: The difference in the average expression level of 42 kinds of markers in $CD45^{+}$ cells between the HC and EH groups. **P* < 0.05, ***P* < 0.01, ****P* < 0.001.

TABLE S2: Markers in EH

Cell type	Marker	Function	Trend	P value
**$CD45^+$**	CD45	CD45 is a receptor protein tyrosine phosphatase, associated with cell development and activation[1].	↑	<0.001
	CD45RO	A marker of activated/memory cells[2].	↑	0.002
	CD11b	CD11b is the α-chain of CD11b/CD18[3], and CD11b/CD18 mediates cellular adhesion via fibrinogen, EPCR, and ICAM-1 on the endothelium[4].	↓	0.002
**Granulocytes**	CD45	Same as above.	↑	0.009
	Ki67	Ki67 is a marker of cell proliferation[5].	↑	0.04
	Foxp3	Foxp3 is a member of the forkhead transcription factor family and has an immunosuppressive effect [6].	↑	0.02
	CD24	CD24 can participate in tyrosine kinase and G protein signaling after adhering to lipid rafts[7].	↑	0.02
	CD31	CD31 is involved in leukocyte diapedesis, angiogenesis, and maintaining the integrity of endothelial cell junctions[8].	↑	0.008
**B cells**	CD45	Same as above.	↑	0.008
	IgM	The binding of IgM to FcμR is essential for the cooperation of B and T cells, the activation of the complement system and ADCC[9].	↑	0.03
	CD19	CD19 is a co-receptor for BCR[10].	↑	0.02
	CD11c	CD11c/CD18 can participate in cell adhesion, migration, and phagocytosis[11].	↓	0.008
	CD45RO	Same as above.	↑	<0.001
	Ki67	Same as above.	↑	0.004
	CXCR3	CXCR3 binds to ligands CXCL9, 10, 11 (mainly secreted by monocytes, fibroblasts and endothelial cells) to regulate cell activation, differentiation and migration[12].	↑	0.02
	CCR7	The CCL19, 21/CCR7 axis mainly helps DCs, T and B cells to migrate to secondary lymphoid organs to activate the immune response[13].	↑	0.02
	CD24	Same as above.	↑	0.008
	CD38	CD38 catabolizes NAD^+ into ADPR and cADPR, which leads to calcium mobilization[14].	↑	0.03
	CD31	In addition to endothelial adhesion mediated by CD31[8], phosphorylation of ITIMs motifs in CD31 activates SHIP, SHP-1 and SHP-2, thereby inhibiting TCR and BCR signaling[15].	↑	<0.001
	CXCR5	CXCL13/CXCR5 promotes the migration of B and T	↑	0.02

		cells to lymphoid follicles[16].		
	HLA-DR	HLA-DR is a key molecule in antigen presentation[17].	↑	0.01
	CD11b	Same as above.	↓	<0.001

Abbreviations: *EPCR* endothelial protein C receptor, *ICAM-1* intercellular adhesion molecule-1, *ADCC* antibody-dependent cell-mediated cytotoxicity, *BCR* B cell receptor, *NAD*$^+$ nicotinamide adenine dinucleotide, *cADPR* cyclic adenosine diphosphate ribose, *ITIM* immunoreceptor tyrosine-based inhibition motif, *SHIP* SH2-containing inositol phosphatase, *SHP* SH2 domain-containing protein tyrosine phosphatase. "↑" represents the increased expression level of marker in EH compared with HC, and vice versa.

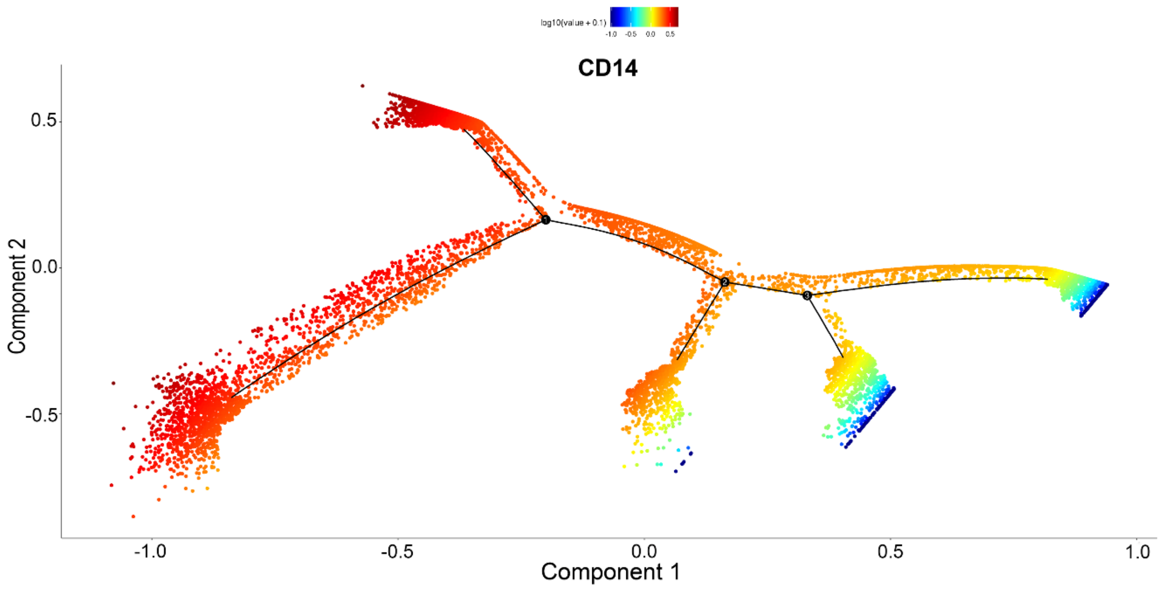

(a)

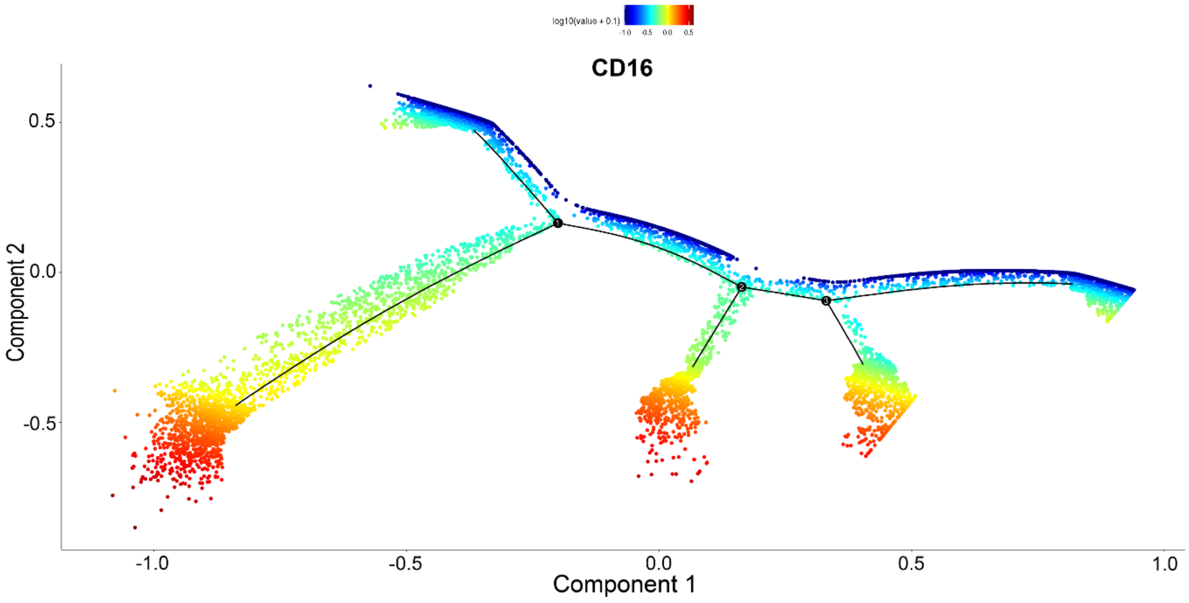

(b)

FIGURE S2: Pseudo-time analysis of monocytes (merge sample). (a) The pseudo-time analysis of CD14. (b) The pseudo-time analysis of CD16. Red represents high expression value and blue represents low expression value.

TABLE S3: The correlation between indexes and blood pressure/age

Index		P value			
		SBP	DBP	PP	Age
Percentage in $CD45^+$ cells	DCs	ns	*	ns	**
	Granulocytes	**	**	ns	ns
	C03	**	**	ns	ns
	C05	ns	ns	ns	**
	C06	*	*	ns	***
	C12	ns	*	ns	ns
	C17	**	**	ns	*
	C21	*	**	ns	*
	C22	*	*	ns	**
	C24	**	**	ns	*
	C25	*	*	ns	*
	C30	*	*	ns	*
Percentage in monocytes	C01	*	**	ns	ns
	C03	*	*	ns	***
	C08	ns	*	ns	ns
	C09	*	**	ns	**
	C10	ns	*	ns	***
	C12	*	*	ns	*
Marker expression in $CD45^+$ cells	CD45	**	**	ns	**
	CD95	ns	*	ns	ns
	CD45RO	*	*	ns	*
	CCR5	ns	ns	ns	ns
	Ki67	ns	*	ns	**
	TLR2	*	**	ns	*
	Foxp3	ns	*	ns	*
	CD38	ns	**	ns	ns
	CD69	ns	*	ns	*
	PD-1	ns	ns	ns	ns
	CD11b	**	*	ns	*
Marker expression in granulocytes	CD45	*	***	ns	*
	Ki67	*	**	ns	ns
	Foxp3	*	*	ns	**
	CD24	**	**	ns	*
	CD31	**	**	ns	*
Marker expression in B cells	CD45	**	*	ns	**
	IgM	ns	ns	ns	ns
	CD19	**	*	ns	ns
	CD11c	*	*	ns	*
	CD45RO	*	**	ns	*

	Ki67	ns	*	ns	*
	CXCR3	ns	*	ns	*
	CCR7	ns	ns	ns	ns
	CD24	*	*	ns	*
	CD38	ns	**	ns	ns
	CD31	*	***	ns	*
	CXCR5	ns	*	ns	ns
	HLA-DR	ns	*	ns	*
	CD11b	**	**	ns	*

References of Supplementary Materials

[1] A. Rheinlander, B. Schraven, U. Bommhardt, CD45 in human physiology and clinical medicine, Immunol Lett, 196 (2018) 22-32.https://doi.org/10.1016/j.imlet.2018.01.009

[2] M.A. Al Barashdi, A. Ali, M.F. McMullin, K. Mills, Protein tyrosine phosphatase receptor type C (PTPRC or CD45), J Clin Pathol, 74 (2021) 548-552.https://doi.org/10.1136/jclinpath-2020-206927

[3] M.A. Arnaout, Structure and function of the leukocyte adhesion molecules CD11/CD18, Blood, 75 (1990) 1037-1050.

[4] L. Martinez, X. Li, G. Ramos-Echazabal, H. Faridi, Z.M. Zigmond, N. Santos Falcon, D.R. Hernandez, S.A. Shehadeh, O.C. Velazquez, V. Gupta, R.I. Vazquez-Padron, A Genetic Model of Constitutively Active Integrin CD11b/CD18, J Immunol, 205 (2020) 2545-2553.https://doi.org/10.4049/jimmunol.1901402

[5] S.S. Menon, C. Guruvayoorappan, K.M. Sakthivel, R.R. Rasmi, Ki-67 protein as a tumour proliferation marker, Clin Chim Acta, 491 (2019) 39-45.https://doi.org/10.1016/j.cca.2019.01.011

[6] J. Vent-Schmidt, J.M. Han, K.G. MacDonald, M.K. Levings, The role of FOXP3 in regulating immune responses, Int Rev Immunol, 33 (2014) 110-128.https://doi.org/10.3109/08830185.2013.811657

[7] S.S. Yin, F.H. Gao, Molecular Mechanism of Tumor Cell Immune Escape Mediated by CD24/Siglec-10, Front Immunol, 11 (2020) 1324.https://doi.org/10.3389/fimmu.2020.01324

[8] J.R. Privratsky, P.J. Newman, PECAM-1: regulator of endothelial junctional integrity, Cell Tissue Res, 355 (2014) 607-619.https://doi.org/10.1007/s00441-013-1779-3

[9] M. Fereidan-Esfahani, T. Nayfeh, A. Warrington, C.L. Howe, M. Rodriguez, IgM Natural Autoantibodies in Physiology and the Treatment of Disease, Methods Mol Biol, 1904 (2019) 53-81.https://doi.org/10.1007/978-1-4939-8958-4_3

[10] X. Li, Y. Ding, M. Zi, L. Sun, W. Zhang, S. Chen, Y. Xu, CD19, from bench to bedside, Immunol Lett, 183 (2017) 86-95.https://doi.org/10.1016/j.imlet.2017.01.010

[11] L.M. Uotila, M. Aatonen, C.G. Gahmberg, Integrin CD11c/CD18 alpha-chain phosphorylation is functionally important, J Biol Chem, 288 (2013) 33494-33499.https://doi.org/10.1074/jbc.C113.497446

[12] R. Tokunaga, W. Zhang, M. Naseem, A. Puccini, M.D. Berger, S. Soni, M. McSkane, H. Baba, H.J. Lenz, CXCL9, CXCL10, CXCL11/CXCR3 axis for immune activation - A target for novel cancer therapy, Cancer Treat Rev, 63 (2018) 40-47.https://doi.org/10.1016/j.ctrv.2017.11.007

[13] R. Tokunaga, M. Naseem, J.H. Lo, F. Battaglin, S. Soni, A. Puccini, M.D. Berger, W. Zhang, H. Baba, H.J. Lenz, B cell and B cell-related pathways for novel cancer treatments, Cancer Treat Rev, 73 (2019) 10-19.https://doi.org/10.1016/j.ctrv.2018.12.001

[14] K.A. Hogan, C.C.S. Chini, E.N. Chini, The Multi-faceted Ecto-enzyme CD38: Roles in Immunomodulation, Cancer, Aging, and Metabolic Diseases, Front Immunol, 10 (2019) 1187.https://doi.org/10.3389/fimmu.2019.01187

[15] F.M. Marelli-Berg, M. Clement, C. Mauro, G. Caligiuri, An immunologist's guide to CD31 function in T-cells, J Cell Sci, 126 (2013) 2343-2352.https://doi.org/10.1242/jcs.124099

[16] C.G. Vinuesa, M.A. Linterman, D. Yu, I.C. MacLennan, Follicular Helper T Cells, Annu Rev Immunol, 34 (2016) 335-368.https://doi.org/10.1146/annurev-immunol-041015-055605

[17] S.A. Erokhina, M.A. Streltsova, L.M. Kanevskiy, M.V. Grechikhina, A.M. Sapozhnikov, E.I. Kovalenko, HLA-DR-expressing NK cells: Effective killers suspected for antigen presentation, J Leukoc Biol, 109 (2021) 327-337.https://doi.org/10.1002/JLB.3RU0420-668RR
